# Age-related changes in human bone marrow mesenchymal stromal cells: morphology, gene expression profile, immunomodulatory activity and miRNA expression

**DOI:** 10.3389/fimmu.2023.1267550

**Published:** 2023-12-07

**Authors:** Fulvio Massaro, Florent Corrillon, Basile Stamatopoulos, Nathan Dubois, Achille Ruer, Nathalie Meuleman, Dominique Bron, Laurence Lagneaux

**Affiliations:** ^1^ Department of Hematology, Jules Bordet Institute, Université Libre de Bruxelles (ULB), Brussels, Belgium; ^2^ PhD Program in Clinical and Experimental Medicine, University of Modena and Reggio Emilia, Modena, Italy; ^3^ Laboratory of Clinical Cell Therapy, Jules Bordet Institute, ULB Cancer Research Center (U-CRC) - Université Libre de Bruxelles (ULB), Brussels, Belgium

**Keywords:** mesenchymal stromal cells, bone marrow, aging, immunomodulation, macrophage polarization

## Abstract

**Introduction:**

Mesenchymal stromal cells (MSC) are one of the main cellular components of bone marrow (BM) microenvironment. MSC play a key role in tissue regeneration, but they are also capable of immunomodulating activity. With host aging, MSC undergo age-related changes, which alter these functions, contributing to the set-up of “inflammaging”, which is known to be the basis for the development of several diseases of the elderly, including cancer. However, there’s few data investigating this facet of MSC, mainly obtained using murine models or replicative senescence. The aim of this research was to identify morphological, molecular and functional alterations of human bone marrow-derived MSC from young (yBM-MSC) and old (oBM-MSC) healthy donors.

**Methods:**

MSC were identified by analysis of cell-surface markers according to the ISCT criteria. To evaluate response to inflammatory status, MSC were incubated for 24h in the presence of IL-1β, IFN-α, IFN-ɣ and TNF-α. Macrophages were obtained by differentiation of THP-1 cells through PMA exposure. For M1 polarization experiments, a 24h incubation with LPS and IFN-ɣ was performed. MSC were plated at the bottom of the co-culture transwell system for all the time of cytokine exposure. Gene expression was evaluated by real-time PCR after RNA extraction from BM-MSC or THP-1 culture. Secreted cytokines levels were quantitated through ELISA assays.

**Results:**

Aging MSC display changes in size, morphology and granularity. Higher levels of β-Gal, reactive oxygen species (ROS), IL-6 and IL-8 and impaired colony-forming and cell cycle progression abilities were found in oBM-MSC. Gene expression profile seems to vary according to subjects’ age and particularly in oBM-MSC seem to be characterized by an impaired immunomodulating activity, with a reduced inhibition of macrophage M1 status. The comparative analysis of microRNA (miRNA) expression in yBM-MSC and oBM-MSC revealed a significant difference for miRNA known to be involved in macrophage polarization and particularly miR-193b-3p expression is strongly increased after co-culture of macrophages with yBM-MSC.

**Conclusion:**

There are profound differences in terms of morphology, gene and miRNA expression and immunomodulating properties among yBM-MSC and oBM-MSC, supporting the critical role of aging BM microenvironment on senescence, immune-mediated disorders and cancer pathogenesis.

## 1 Introduction

Mesenchymal stromal cells (MSC) are multipotent cells which were firstly isolated from the bone marrow (BM) in the 1960s (1). Their presence has also been described in several other tissues in adults such as umbilical cord, placenta, cartilage, connective tissue, trabecular bone, adipose tissue, endometrium, skin (2–8). The International Society for Cell & Gene Therapy (ISCT) identified three criteria for the identification of MSC: adherence to plastic in standard culture conditions, positivity for three specific surface markers (CD73, CD90, CD105) and absence of hematopoietic antigens (CD45, CD34, CD14, CD79a and HLA-DR), a multipotent differentiation potential (osteoblasts, chondrocytes, adipocytes) (9).

This group of progenitor cells comprises different entities characterized primarily by their capacity of self-renewal and multilineage differentiation potential, presenting minor functional variations according to the source (10–14).

Due to these properties and minor ethical issues, MSC are considered as a promising therapeutical option in the context of regenerative medicine (5, 15). Several studies, with many phase 1/2 studies conducted in humans, showed a promising beneficial effect from the use of MSC in the field of inflammatory and degenerative disorders (16). Until 2008, MSC employed for clinical purposes were almost entirely derived from bone marrow (BM-MSC), while today there is an equal use of BM-, adipose tissue (AT-) and perinatal tissue (PT)-MSC (17). The diversification of MSC products requires thus complementary criteria focused on MSC potency and safety. For example, MSC from different tissue sources display high variability in tissue factor (TF) expression which is the major determinant of cell product hemocompatibility.

There is increasing evidence about the ability of MSC to modulate immune response, both innate and adaptive, particularly in response to environmental stimulation. MSC can limit B and T cell proliferation, dendritic cell differentiation, promote regulatory T cell expansion and M2 macrophage polarization (18).

The mechanisms leading to this function are multiple, involving mainly a relevant paracrine activity characterized by the production of cytokines, chemokines, growth factors and microRNA (miRNA), and to a lesser extent cell-to-cell interactions. Furthermore, the important paracrine activity exerted by MSC is characterized by the production of extracellular vesicles (EVs), which are membrane-encapsulated nanoparticles (size range 30-1000 nm) that play a key role in cell-to-cell interaction and maintaining tissue homeostasis (19–23).

With aging, changes in MSC include loss of differentiation potential, decreased proliferation and an increasing number of senescent cells (24). Senescent MSC undergo major changes at both morphological and functional levels, consequence of genetic and epigenetic variations, such as increased autophagy, mitochondrial disturbances, altered secretory profile (senescence-associated secretory profile; SASP), gene expression modifications and reduced capacity of maintaining tissue homeostasis (25–27).

In animal models, biological aging of BM-MSC induces changes in cellular proliferation, differentiation, clonogenicity, senescence, oxidative stress, DNA damage repair and telomerase shortening (28–31). Several studies in humans have reported a decline in the frequency of CFU-F with the biological age of the BM (32). Aging alters BM-MSC differentiation potential with a loss of osteogenic potential and a gain of adipogenic potential (33). BM-MSC aging is also characterized by several other factors including telomere shortening, oxidative stress and less dynamic cytoskeletal remodeling (34).

Immunomodulation seems to be impaired in aging MSC, but few data exist in this field and they result mainly from murine models or *in vitro* experiments on replicative senescence (27, 35). MSC from young donors have enhanced anti-inflammatory properties. In contrast, “old” MSC tend to exhibit pro-inflammatory features while their immunosuppressive capacity is impaired (36). BM-MSC from younger donors led to lower IL-6 production when co-cultured with activated T cells while aged MSC were shown to inhibit T cells less efficiently, being also associated with a decreased indoleamine 2,3-dioxygenase (IDO) activity in response to inflammation (37). This impaired immunomodulatory ability seems to play a role in supporting the “inflammaging” process, a state of mild-grade chronic pro-inflammatory condition which is associated to senescence and favorizes degenerative disorders typically found in the elderly (38). Senescent MSC could contribute to this process through the secretion of pro-inflammatory cytokines and EVs, impaired macrophages M2 polarization and an aberrant behavior inside the hematopoietic niche (39).

The impact of aging on the immunomodulatory properties of MSC, particularly their regulatory effect on macrophages, remains largely unknown. Since MSC can promote the polarization of macrophages toward an anti-inflammatory/immune-regulatory (M2) phenotype, we aim to evaluate the impact of donor age on this function. Inflammaging is a macrophage-centered phenomenon and given that senescent MSC promote myeloid cell generation and innate immune activation, we hypothesized that MSC aging could also affect macrophage polarization (40).

We here report results from our study, whose purpose was to identify morphological, molecular and functional alterations of BM-MSC from healthy young (yBM-MSC) and old (oBM-MSC) donors and particularly the impact of MSC aging on macrophage polarization.

## 2 Materials and methods

### 2.1 Isolation, culture and expansion of BM-MSC

Bone marrow samples were harvested from the sternum or the posterior iliac crest of healthy volunteers after administration and signature of an informed consent. In some cases, we obtained cells from the washouts of discharged bags and filters of hematopoietic stem cell transplantation as previously described (41). We defined young and old donors as subjects having less than 18 years or more than 55 years, respectively. The **Table 1** displays the age distribution among the two groups. MSC were isolated using the classical adhesion method. Mononuclear cells were isolated by density gradient centrifugation (Pancoll, Palm Biotech, Aidenbach, Deutschland), washed in Hank’s buffered salt solution (HBSS, Lonza Europe, Verviers, Belgium) and seeded in Dulbecco’s modified Eagle’s medium–low glucose (DMEM-LG, Lonza) supplemented with 15% of fetal bovine serum (FBS, Sigma-Aldrich, Saint-Louis, MO, USA), 2 mM L-glutamine and 1% penicillin-streptomycin (both from Lonza). Cells were incubated at 37°C in a 5% CO2 and 95% humidified atmosphere. After 48 h, non-adherent cells were removed by washing and the medium was changed twice a week. Thereafter, cells were detached with TrypleSelect solution (Lonza) and subcultured at 10^3^ cells/cm^2^ for their expansion by passage. MSC were immunophenotypically characterized according the ISCT criteria and their adipogenic, osteogenic and chondrogenic differentiation potential evaluated (9).

**Table 1:** Age distribution for donors included in our study.

Young donors (n=28)	Old donors (n=20)
1	55
2	56
3	56
3	57
3	57
3	57
4	58
4	61
4	61
5	61
6	63
6	65
7	65
7	66
8	68
8	69
8	69
10	73
10	77
13	92
14	
15	
15	
16	
16	
16	
17	
17	
**9 ± 1**	**64 ± 2**

### 2.2 Characterization of BM-MSC

Briefly, MSC immunophenotype was established by flow cytometry using the following monoclonal antibodies: anti-CD73-PE (Miltenyi Biotec, Bergisch Gladbach, Germany), anti-CD90-PE (Miltenyi Biotec), anti-CD105-FITC (Ancell Corporation, Bayport, USA), anti-CD45-PC7 (BD Biosciences), anti-CD34-PE (Miltenyi), anti-CD14-PC7 (BD Biosciences), CD11b-APC (Miltenyi Biotec) and CD19-PC5 (BD Biosciences). After labeling, acquired results were analyzed by using a MACSQuant analyzer (Miltenyi Biotec). Cells were incubated for 30 min with these antibodies and after washing with PBS, the cells were fixed with 8% formaldehyde. BM-MSC were cultured in appropriate induction medium to assess their adipogenic, osteogenic and chondrogenic lineage differentiation capacities (NH media, Miltenyi Biotec). Lipid vacuole formation, mineralization (calcium deposits) and presence of proteoglycans corresponding to each lineage commitment were demonstrated by Oil Red O (Sigma-Aldrich), Alizarin Red S (Sigma-Aldrich) and Alcian Blue (Sigma-Aldrich) staining respectively.

### 2.3 Population doubling time

Population doubling time (PDT) was calculated between P1 and P2 as t/n, where t is the duration of culture in days and n is the number of population doublings calculated by using the formula n = (log Nh - log Ni)/log 2, where Nh is the number of cells harvested at the time of counting at P2 and Ni is the number of cells initially plated at P1.

### 2.4 Detection of cell senescence

Senescence was evaluated by two methods: β-Galactosidase (β-Gal) staining and flow cytometry using a fluorescent probe. Ten thousand BM-MSC cells were plated in a 48 well-plate for 24 h before staining cells with the Senescence Detection Kit (BioVision, Milpitas, CA, USA). Blue cells were observed using an inverted microscope and the number of blue cells out of 100 total cells was scored. For the second method, cells were incubated with diluted Green probe (1000x) for 2 hours at 37°C without CO2 (Invitrogen CellEvent™Senescence Green detection kit). After incubation, cells were washed with PBS and analyzed by flow cytometry (MACSQuant, AlexaFluor™ 488/FITC filter set). The results were presented as the mean percentage (± SEM) of senescent cells.

### 2.5 Intracellular ROS detection

Intracellular reactive oxygen species (ROS) were measured by a DCFDA fluorescent probe (Sigma-Aldrich). Briefly, young and old BM-MSC were wash with PBS twice and incubated with serum-free DMEM containing 10 µM DCFDA at 37°C for 20 min. ROS generation was determined by flow cytometry and the percentage of positive cells and the mean fluorescence intensity (MFI) in each group was evaluated. A shift to the right indicates increased ROS levels.

### 2.6 Clonogenic assay

To estimate the number of mesenchymal clonogenic cells, a colony-forming unit fibroblast (CFU-F) assay was performed. Briefly 5000 cells were plated in a Petri dish (100 mm diameter, Greiner) with culture medium for 10 days. After May-Grünwald/Giemsa staining, colonies were defined as more than 50 fibroblastic cells and scored using an inverted microscope.

### 2.7 Cell cycle analysis

Cell cycle was evaluated by standard propidium iodide (PI) staining. Cells to be analyzed were collected, washed, permeabilized, and incubated with solution containing PI and RNAse (Coulter DNA-Prep Reagent, Hialeah, FL, USA). Tubes were placed at 4°C in the dark overnight before analysis by flow cytometry to identify the cell cycle phases. Data collection was gated using forward light scatter and side light scatter to exclude cell debris and aggregates. PI fluorescence of individual nuclei was measured using MACSQuant flow cytometer and a least 2 10^4^ cells of each sample were analyzed by FCS Express 4 Flow software.

### 2.8 Inflammatory conditions

The impact of an inflammatory environment on BM-MSC was evaluated as previously described (42). Briefly, cells were stimulated for 24h using a cocktail of pro- inflammatory cytokines: 25 ng/mL IL-1β (Miltenyi Biotec), 50 ng/mL interferon (IFN)-γ (RD Systems, Abingdon, United Kingdom), 50 ng/mL tumor necrosis factor alpha (TNF-α) (Miltenyi Biotec), and 10 ng/mL IFN-α (RD Systems).

### 2.9 Polarization of macrophages and co-cultures with BM-MSC

Human monocytic THP-1 cells were maintained in culture in RPMI 1640 culture medium (Biowest, Nuaillé, France) containing 10% of heat inactivated fetal bovine serum (Sigma). THP-1 monocytes were differentiated into macrophages by 24 h incubation with 200 ng/ml phorbol 12-myristate 13-acetate (PMA, Sigma). Macrophages were polarized in M1 macrophages by incubation with 20 ng/ml of IFN-γ (R&D systems) and 100 ng/ml of LPS (Sigma, #8630). Macrophage M2 polarization was obtained by incubation with 20 ng/ml of interleukin-4 (Tebu-Bio, Boechout, Belgium) and 20 ng/ml of interleukin-13 (Tebu-Bio).

BM-MSC and THP-1-derived macrophages were co-cultured using a cell culture insert (Greiner Bio-One, Vilvoorde, Belgium) with a 0.4-μm porous membrane to separate the upper and lower chambers. The THP-1 monocytes (1 × 10^5^ cells/ml) were seeded into the lower chamber of the Transwell apparatus (12 well plates), stimulated to differentiate into macrophages by the addition of PMA during 24h. BM-MSC were placed in the upper chamber at a density of 1 × 10^5^ cells/ml for 24 h to allow their adherence to the membrane. The chambers with the BM-MSC were then placed directly on top of the 12-well plates containing the THP-1-derived macrophages and the resulting co-culture systems were incubated for 24 h. Co-culture supernatants were then collected to quantitate cytokine levels and THP-1 derived macrophages were harvested for mRNA and flow cytometry assessment of specific cell surface antigens.

### 2.10 Quantitative real-time PCR

Total RNA was isolated from BM-MSC or THP-1 and was extracted in a single step using TriPure Isolation Reagent (Roche Applied Science, Vilvoorde, Belgium). We performed the reverse transcription reaction with 1 mg RNA using qScript cDNA SuperMix (Quanta Biosciences). Transcripts were quantified by qRT-PCR using 20 ng of cDNA, SYBR Green PCR Master Mix (Applied Biosystems, Lennik, Belgium) and 0.32 mM forward and reverse primers. The primers were designed with Primer Express 2.0 software (Applied Biosystems) or ProbeFinder online software (Roche) and are available in **Table 2**. To control variations in input RNA amounts, the *GAPDH* gene was used as a housekeeping gene to quantify and normalize the results. The reactions were carried out using the ABI Prism 7900 HT system (Applied Biosystems). The comparative ΔΔCt method was used for data analysis. To evaluate the fold change, data were normalized with the *GAPDH* gene to obtain the ΔCt and were then calibrated with the geometric mean of the *GAPDH* ΔCt to generate the ΔΔCt. Fold changes were then calculated as fold change=2–^ΔΔCt^.

**Table 2:** List of primer sequences used in RT-PCR.

Gene	Forward	Reverse
p16	TGCCTITCACTGTGTTIGGA	TGCTTGTCATGAAGTCGACAG
p21	CGAAGTCAGTTCCTTGTGGAG	CATGGGTTCTGACGGACAT
p53	AGGCCTTGGAACTCAAGGAT	CCCTIITTGGACTTCAGGTG
COX-1	CCTGCAGCTGGAAATTTGACCCA	ACCTTGAAGGAGTCAGGCATG
COX-2	GCTAAACATGATGTTTGCATTO	GCTGGCCTCGCTTATGA
GALL	AAGCTGCCAGATGGATACGAA	CGTCAGCTGCCATGTAGTTGA
HGF	CAATGCCTCTGGTTCCCCTT	AGGCAAAAAGCTGTGTTCGTG
IDO1	TTCAGTGCTTTGACGTCCTG	TGGAGGAACTGAGCAGCAT
UF	TGAAAACTGCCGGCATCTGA	CIGIGTACTGCCGCCAAGA
TSG-6	TCATGTCTGTGCTGCTGGATG	GGGCCCTGGCTTCACAA
IL-1β	AGTGGTGTTCTCCATGTCCTTIGTA	GCCCAAGGCCACAGGTATT
IL-6	AAATTCGGTACATCCTCGACGG	GGAAGGTTCAGGTTGTTTICTGO
IL-8	CTGTTAAATCTGGCAACCCTAGICT	CAAGGCACAGIGGAACAAGGA
IL-10	AGAACCTGAAGACCCTCAGGO	CCACGGCCTTGCTCTIGTT
TNF-α	ATCTTICTCGAACCCCGAGIGA	AGCIGCCOCTCAGCTIGA
CC1-2	ATCAATTGCCCCAGTCACO	AGTCTTCGGAGTTIGGG
TGM2	GCCACTTCATIIIGCTCTIC	TOCTCTTCCGAGTCCAGGTTACA
ARG1	CAGAGCATGAGCGCCAAGT	TOGTGGCTGTCCCTITGAG
GAPDH	AATCCCATCACCATCTTCCA	TGGACTCCACGACGTACTCA

MiRNA were quantified by real-time PCR using SYBR green. Briefly, miRNA will be first polyadenylated using a poly(A) polymerase. Reverse Transcription will be performed using an oligo-dT adapter primer. The adapter primer (GCATAGACCTGAATGGCGGTA) has a unique sequence at its 5’ end which allows amplification of cDNA in real-time PCR reactions. Individual miRNA were quantified in real-time SYBR Green PCR with the desired customer miRNA complementary to the target miRNA (miR-193b: GCCCTCAAAGTCCCGCTAA, miR-21: GCAGCTTATCAGACTGATGTTGAAA) and with a specific primer to the unique sequence of the oligo-dT adapter primer. The custom miRNA provides maximum sensitivity and specificity in real-time PCR amplification and quantification of miRNA. Cell miRNA will be normalized using RNU44 (CCTGGATGATGATAGCAAATGC) and RNU48 (CTCTGAGTGTGTCGCTGATGC), largely described as stable endogenous controls (43).

### 2.11 Enzyme-linked immunosorbent assay

Secreted protein levels of CCL2, TNF-α and IL-6 were quantitated in culture supernatants of THP-1 derived macrophages co-cultured or not with BM-MSC, in the presence or not of M1 activation using specific cytokine ELISA kits (Quantikine, RD Systems) as per manufacturer’s instructions. The sensitivity of CCL2, TNF-α and IL-6 was respectively 10, 6.23 and 2.95 pg/ml. In some experiments, culture supernatants were analyzed for cytokine levels by fluorescent flow cytometry using the Bio-Plex Pro Human Cytokine Panel, 48-plex (Bio-Rad, USA). The assay was performed using a Bio-Plex machine (Bio-Plex 200 System, Bio-Rad) following the manufacturer’s protocol, and the data were analyzed with the Bio-Plex manager software version 6.0.

### 2.12 Statistical analysis

Data are presented as mean ± standard error of the mean (SEM). Comparison between data was evaluated with the unpaired Mann-Whitney U test. In some cases, the Wilcoxon matched pair test (two-tailed) was used and differences were significant for p< 0.05. All analyses were performed with GraphPad Prism version 5.00 for Windows (GraphPad Software, www.graphpad.com).

## 3 Results

### 3.1 MSC identification, morphology, differentiation potential and cell cycle progression

**Figure 1:**
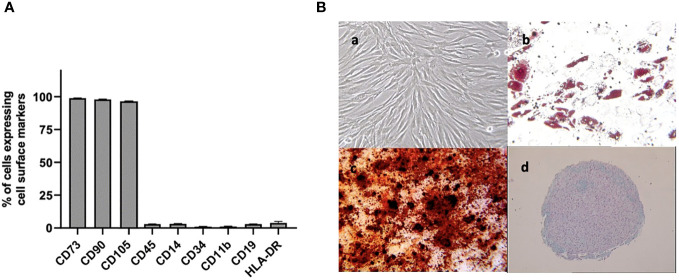
**(A)** Cell surface marker expression on BM-MSC at passage 2, evaluated by flow cytometry (n=3) **(B)** Analysis of cell differentiation potential of MSC at passage 2: (a) undifferentiated MSC observed by phase contrast microscopy (magnification x100). After 2-3 weeks in respective induction media, MSC were stained positively for lipid vacuoles with Oil Red O (b)(x100) showed the formation of mineralized matrix assessed by Alizarin red staining (x100) (c) and were positive for glycosaminoglycan matrix stained by Alcian blue (d) (x25) indicating adipogenic, osteogenic and chondrogenic differentiation respectively.

**Figure 2:**
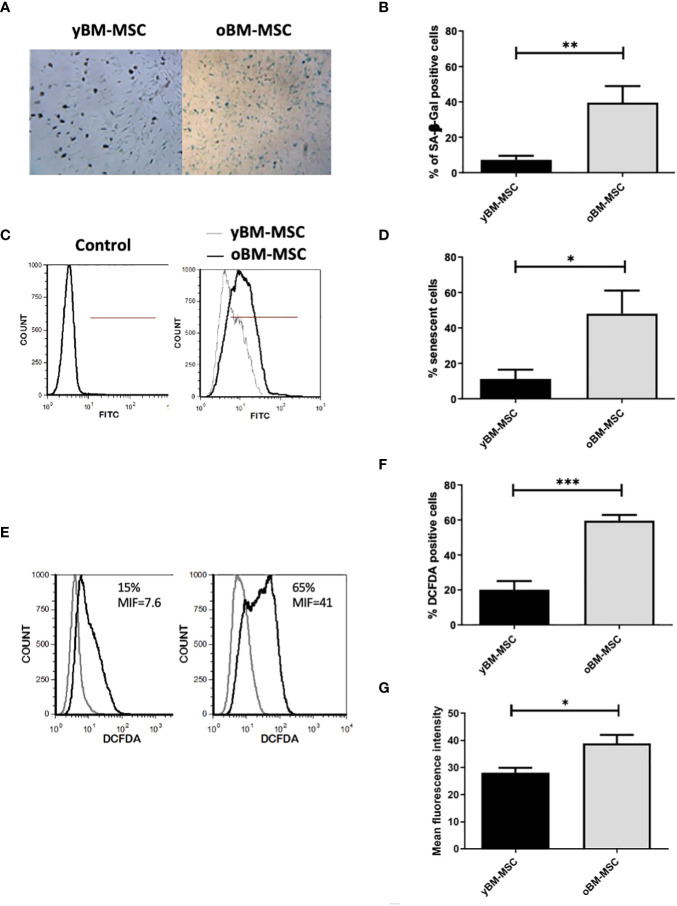
Increased senescence of oBM-MSC and ROS generation in comparison with yBM-MSC at P2. **(A)** yBM-MSC and oBM-MSC were stained for senescence-associated β-Galactosidase (SA β-Gal) and **(B)** the number of blue cells out 100 total cells in the well was scored using an inverted microscope. The results were presented as the mean percentage (± SEM) of blue cells compared to total counted cells (n= 5; **p<0.02). **(C)** Representative flow cytometry histograms showing senescent cells detected using a fluorescent probe. The percentage of positive cells was determined in comparison to the unlabeling cells representing negative control. **(D)** Percentage of senescent cells evaluated by flow cytometry for y-BM-MSC and oBM-MSC (n=5;* p<0.04) **(E)** Representative flow cytometry of ROS detection **(F)** Percentage of DCFDA positive cells and **(G)** Mean fluorescence intensity after DCFDA labeling (yBM-MSC, n= 8 and oBM-MSC, n=20; *p<0.03 ***p<0.0001).

**Figure 3:**
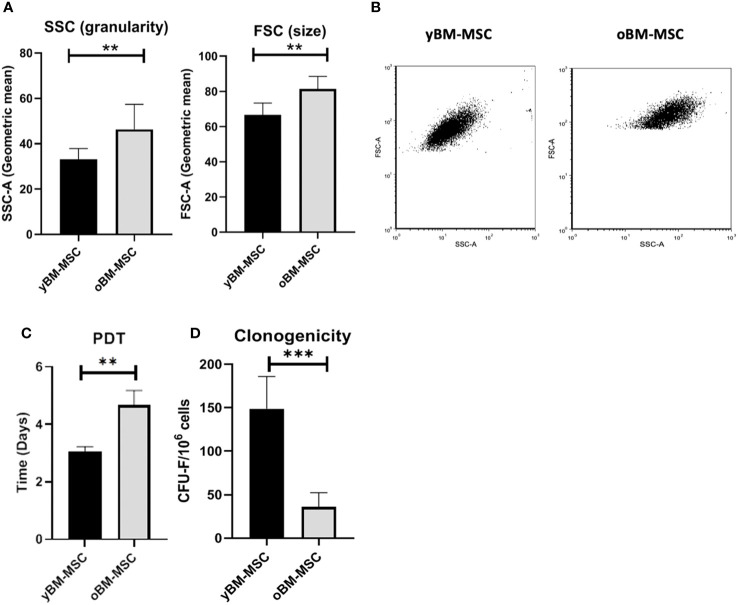
**(A)** Cell size and granularity of yBM-MSC (n=8) and oBM-MSC (n=8) evaluated by flow cytometry. **(B)** Representative flow cytometry dot plots showing increased size and granularity in oBM-MSC. **(C)** Population doubling time of yBM-MSC (n=10) and oBM-MSC (n=10) cultures calculated between P1 and P2. **(D)** Clonogenicity of yBM-MSC (n=28) and oBM-MSC (n=16) evaluated by CFU-F assay at P1. **p<0.005, ***p<0.0003.

**Figure 4:**
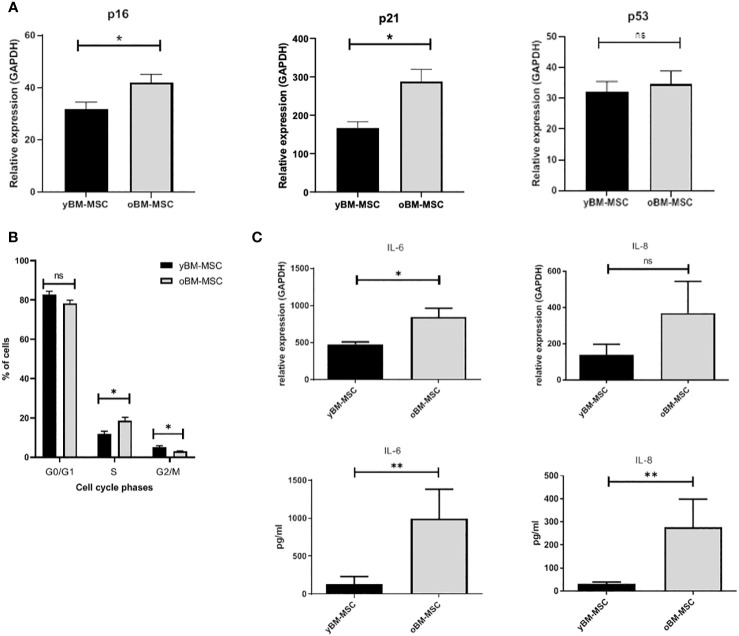
**(A)** Expression of cell cycle regulators p16, p21 and p53 in yBM-MSC and oBM-MSC, evaluated by RT-PCR (n=11). **(B)** Cell cycle distribution of yBM-MSC and oBM-MSC determined by flow cytometry after propidium iodide DNA staining (n=8). **(C)** Increased expression of IL-6 and IL-8 at mRNA (n=22 and n=15) and protein levels (n=9 and n=8) in oBM-MSC in comparison to yBM-MSC. * p<0.03, **p<0.008, ns=not significant.

A flow cytometry assay conducted after MSC isolation and expansion showed the co-expression of CD73, CD90 and CD105 in more than 98% of analyzed cells, while the expression of hematopoietic markers CD45, CD14, CD34, CD11b, CD19 and HLA-DR was inferior to 2%, compatible with International Society for Cellular Therapy (ISCT) criteria for MSC identification (**Figure 1A**). Both yBM- and oBM-MSC were capable of adipogenic, osteogenic and chondrogenic differentiation (**Figure 1B**). β-Gal staining showed a significantly increased percentage of senescent cells for oBM-MSC compared to yBM-MSC (39.6 ± 9.4 vs 7 ± 2.4, n=5; *p*=0.016) (**Figures 2A, B**). These results were confirmed by flow cytometry (47% ± 14% vs 10% ± 6%, n=5; *p*=0.046) (**Figures 2C, D**). DCFDA staining showed that 20 ± 5% of yBM-MSC and 60 ± 3% of oBM-MSC were ROS positive (*p*<0.0001) (**Figures 2E, F**) with an increased level of intracellular ROS for oBM-MSC (MFI of 38,85 ± 3.17, n= 20) compared with yBM-MSC (MFI of 28 ± 1.7, n= 8, *p*= 0.027) (**Figure 2G**). Concerning morphology, oBM-MSC showed significantly increased size (81.38 ± 2.51 vs 66.62 ± 2.38; *p*=0.005) and granularity (46.35 ± 3.9 vs 33 ± 1.7; *p*=0.007) compared to yBM-MSC (**Figures 3A, B**). Mean population doubling time was significantly longer for oBM-MSC compared to yBM-MSC (4.68 ± 0.5 vs 3.04 ± 0.28 days; *p*=0.001) (**Figure 3C**). We observed a reduced clonogenic capacity of oBM-MSC (n= 8) compared to yBM-MSC (n= 16) (36 ± 16.5 colonies vs 149 ± 37 colonies for 10^6^ cells; *p*=0.0001) (**Figure 3D**). The analysis of the expression of cell cycle regulators showed significantly higher levels in oBM-MSC for *p16* (41.78 ± 3.34 vs 31.67 ± 2.71; *p*=0.03) and *p21* (287.7 ± 32.03 vs 166.4 ± 16.46; *p*=0.01), while no difference was shown for *p53* (34.64 ± 4.32 vs 32.11 ± 3.4) (**Figure 4A**). The evaluation of cell cycle phase showed a higher number of oBM-MSC in the S phase (18.72 ± 1.71 vs 11.91 ± 1.38; *p*=0.02) and a lower number in the mitotic phase (3.05 ± 0.28 vs 5.16 ± 0.69; *p*=0.03) (**Figure 4B**). At mRNA and protein levels, we observed a higher expression and secretion of IL-6 and IL-8 by oBM-MSC compared to yBM-MSC. The relative IL-6 and IL-8 gene expression was respectively 477 ± 34.6 vs 846 ± 119 (n= 22; *p*=0.0267) and 224 ± 58 vs 369 ± 174 (n= 15; *p*=0.1). By ELISA, we confirmed higher IL-6 and IL-8 production by oBM-MSC compared to yBM-MSC (respectively 130 ± 101 vs 989 ± 394 pg/ml; *p*=0.0076 for IL-6 (n=9) and 224 ± 58 vs 369 ± 174; *p*=0.0016 for IL-8 (n= 8) (**Figure 4C**). These results were consistent with the presence of SASP in oBM-MSC.

### 3.2 Gene expression profile after proinflammatory priming

**Figure 5:**
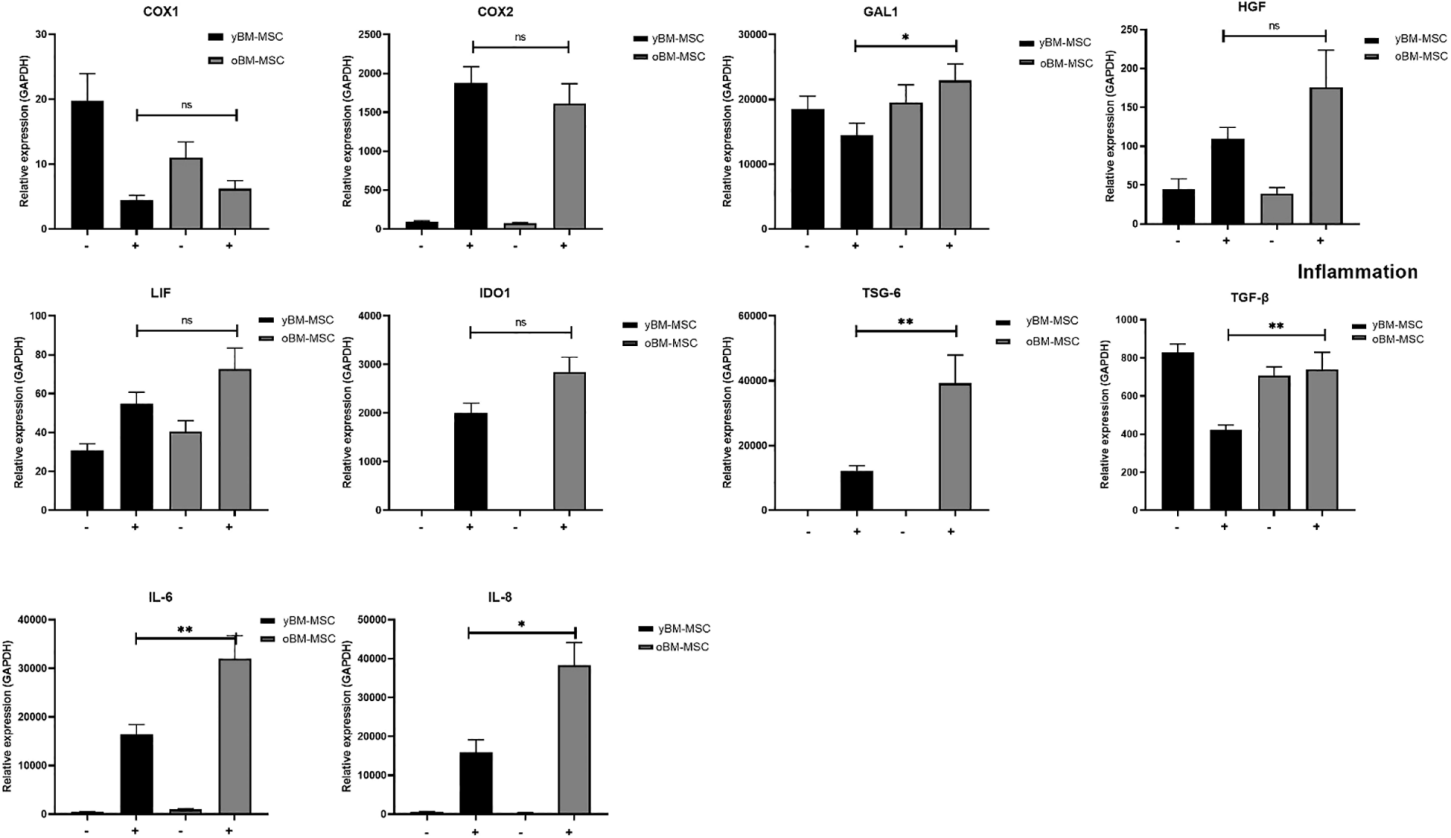
Gene expression profile of yBM-MSC (n=12) and oBM-MSC (n=12) before (–) and after (+) proinflammatory priming, evaluated by RT-PCR. * p<0.05, ** p<0.005, ns=not significant.

We evaluated the variation in mRNA expression for genes implicated in immunomodulation, before and after exposing MSC to a proinflammatory priming, as described in “Material and methods” section. The cocktail was chosen by our group and others because these cytokines are those mostly present at inflammatory sites. Before exposing yBM-MSC and oBM-MSC to stimulating cytokines, we did not report any difference in the two groups in terms of gene expression for the analyzed markers, except for IL-6 and IL-8 (as shown in the previous section). Inflammation modulates several biological features of MSC (secretome, multilineage potential, immunomodulation) and allows to evaluate the MSC efficiency. Indeed, after the priming, we reported a significant up-regulation in oBM-MSC, when compared to yBM-MSC, for galectin-1 (*GAL-1*; 22915 ± 2558 vs 14511 ± 1838; *p=*0.03), interleukin-6 (*IL-6*; 28622 ± 4203 vs 19564 ± 2938; *p*=0.004), interleukin-8 (*IL-8*; 29574 ± 5334 vs 17696 ± 2549; *p*=0.04), transforming growth factor beta (*TGF-β*; 739 ± 91 vs 424 ± 24; *p*=0.003) and tumor necrosis factor stimulating gene-6 (*TSG-6*; 39239 ± 8658 vs 12221 ± 1606; *p*=0.002). For other genes, such as cyclooxygenase-1 (*COX-1*; 6 ± 1.2 vs 4.3 ± 0.69; *p*=0.569) and -2 (*COX-2*; 1619 ± 249 vs 1881 ± 207; *p*=0.470), hepatocyte growth factor (*HGF*; 176 ± 48 vs 110 ± 15; p=0.301), indoleamine 2,3-dioxygenase (*IDO1*; 2845 ± 306 vs 2006 ± 201; *p*=0.176) and leukemia inhibitory factor (*LIF*; 73 ± 11 vs 55 ± 5.7; *p*=0.233), no significant differences were observed (**Figure 5**).

### 3.3 Co-culture and impact of MSC on THP-1 derived macrophage polarization

**Figure 6:**
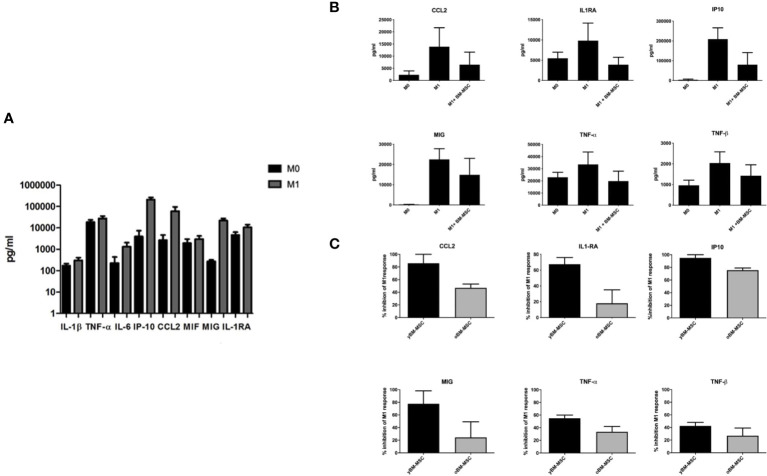
Cytokine levels in culture supernatants of THP-1 macrophages co-cultured or not with BM-MSC for 24 hours. **(A)** Concentration of selected cytokines in THP-1 macrophage culture media, measured at baseline (M0) and after induction of M1 polarization with Bio-Plex Multiplex Immunoassay using the Bio-Plex Pro Huma cytokine panel, 48-plex (n=4). **(B)** Concentration of selected cytokines in THP-1 macrophage culture media before and after co-culture of M0 and M1 macrophages with BM-MSC (n=4). **(C)** Comparison of M1 inhibitory activity exerted by yBM-MSC and oBM-MSC, expressed as percentage reduction of cytokine concentration in THP-1 M1 macrophage culture media before and after co-culture with BM-MSC (n=4).

Through a bio-plex multiplex analysis we identified, in macrophage culture medium, several cytokines with significantly increased concentration after polarization to M1 status (**Figure 6A**). Co-culture of MSC with M1 polarized THP-1 derived macrophages was associated to a decrease of M1 cytokine production (n=4) (**Figure 6B**). We compared the inhibitory activity exerted by yBM- and oBM-MSC through the evaluation of the concentration of chemokine ligand 2 (CCL2), tumor necrosis factor-α (TNF-α), human interferon-inducible protein 10 (IP10), IL-6, monokine induced by interferon gamma (MIG): we found a significantly higher anti-M1 activity, determining a reduction for these cytokines, after adding in co-culture yBM-MSC (**Figure 6C**).

**Figure 7:**
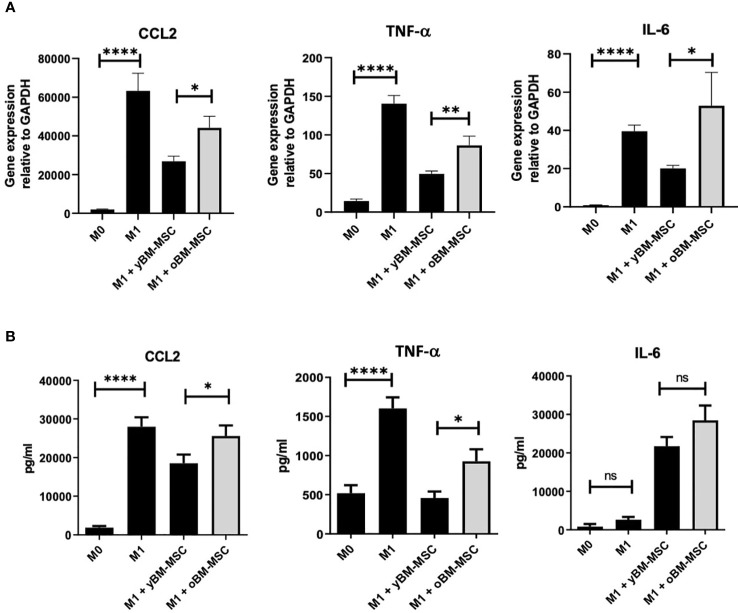
**(A)** Relative gene expression of CCL2 (n=12), IL-6 (n=12) and TNF-α (n=14) in THP-1 macrophages, measured at baseline (M0) and after induction of M1 polarization. Comparison of M1 inhibitory activity exerted by yBM-MSC and oBM-MSC in THP-1 M1 macrophages before and after co-culture with BM-MSC (n=13). **(B)** ELISA assay measuring the concentration of CCL2, TNF-α and IL-6 in THP-1 macrophage culture media at baseline (M0), after induction of M1 polarization, before and after co-culture of M1 macrophages with yBM-MSC and oBM-MSC (n=20). * p<0.05, ** p<0.005 ****p<0.0001, ns=not significant.

We compared the inhibitory effect towards macrophage polarization exerted by yBM-MSC and oBM-MSC, evaluating the mRNA expression of three target cytokines involved in M1 response: *CCL-2*, *TNF-α* and *IL-6*. The relative gene expression of these cytokines increased after M1 polarization of THP-1 macrophages: 1941 ± 290 vs 63296 ± 9051 for CCL2 (*p*<0.0001), 14 ± 2 vs 141 ± 10.5 (*p*<0.0001) for TNF-α and 0.8 ± 0.25 vs 39.6 ± 3.2 for IL-6 (*p*<0.0001) (**Figure 7A**) corresponding to an increase of 59.08 ± 29.42 fold, 10 ± 2.7 fold and 48.45 ± 17.26 fold respectively for CCL2, TNF-α and IL-6. When comparing the impact on the mRNA expression, we reported a significantly reduced expression after co-culture with yBM-MSC if compared to oBM-MSC for *CCL-2* (26809 ± 7135 vs 44146 ± 20886, *p*= 0.04) and *TNF-α* (50 ± 3.6 vs 86 ± 12, *p*= 0.0019). yBM-MSC inhibited CCL2 expression of 54 ± 6.8% and 68 ± 3% for TNF-α while the inhibitory activity by oBM-MSC only reached 34 ± 6% and 39 ± 7%. For *IL-6*, we reported a reduction for yBM-MSC (20.09 ± 1.6 in comparison to 39.6 ± 3.2 for M1 condition corresponding to an inhibition of 52 ± 4%) and an augmentation after oBM-MSC co-culture (52.88 ± 17.5 vs 39.6 ± 3.2; *p*= 0.014).

We also investigated and compared variations in medium concentration for these three cytokines through an ELISA assay. Concentrations of CCL-2 (27974 ± 2435 pg/ml vs 1889 ± 391 pg/ml; *p*< 0.0001) and TNF-α (1603 ± 142 pg/ml vs 519 ± 101 pg/ml; *p*< 0.0001) were significantly higher for M1 macrophages if compared to M0, while for IL-6 we did not find a significant difference (2628 ± 728 vs 812 ± 700 pg/ml) (**Figure 7B**). Concerning the impact of MSC co-culture on these M1 markers, yBM-MSC were associated to a higher percentage reduction in CCL-2 (25% ± 9% vs 20% ± 5.7%, *p*= 0.01) and TNF-α (65% ± 3.35% vs 40.59% ± 6.4%, *p*=0.01) levels. For IL-6 we reported increased values after co-culture with MSC, with non-significantly higher levels for oBM-MSC compared to yBM-MSC (1082% ± 148% vs 828% ± 89%; *p*=0.393).

### 3.4 Analysis of miRNA expression in MSC

**Figure 8:**
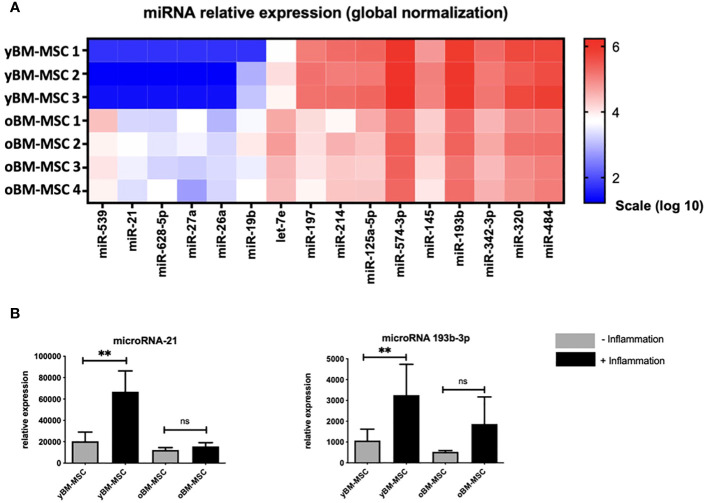
**(A)** Comparison of miRNA expression profile between yBM-MSC (n=3) and oBM-MSC (n=4). **(B)** Comparison of miR21 and miR193b-3p expression for yBM- and oBM-MSC, before and after inflammatory priming (n=11). ** p<0.005, ns=not significant.

We performed a miRNA expression profile analysis comparing yBM- and oBM-MSC, by using selected miRNA previously identified with a potential role in the immunomodulating abilities exerted by MSC and particularly in macrophage polarization (**Figure 8A**). After this preliminary phase, we performed further investigations on two miRNA, miR21 and miR193b-3p: we found a significantly increased relative expression after inflammatory priming for both miR21 (1310 ± 495.2 vs 554 ± 114; *p*=0.001) and miR193b-3p (31844 ± 7967 vs 14748 ± 3044; *p*=0.001) in yBM-MSC, while no significant differences were noted for oBM-MSC (miR21: 723.1 ± 56.56 vs 622.5 ± 57.82; *p*=0.577; miR193b-3p: 16376 ± 1960 vs 15431 ± 2369; *p*=0.243) (**Figure 8B**).

## 4 Discussion

MSC are one of the main components of BM microenvironment, promoting tissue homeostasis and the development of a well-balanced hematopoiesis through their significant secretory activity. Morphological and functional changes occurring in MSC with aging, and particularly the modifications of immunomodulatory activity, have not yet been fully understood and deserve more investigations. In this research work we analyzed human-derived BM-MSC by performing a comparison between two groups of donors of less than 18 years and more than 55 years. These cut-offs have been setup arbitrarily, in the absence of clear discriminant criteria to define young and old subjects and in order to avoid bias and overlap in both populations as already seen in other similar publications (44–46).

We reported important differences in the two examined groups for what concerns morphology, cell cycle progression, expression of senescence markers and ROS generation. The increased expression of β-Gal is a confirmation of the different profile acquired by oBM-MSC: this hydrolase can be found in lysosomes of aging cells at pH 6 while it is not detected in normally proliferating ones and for this reason it is considered as a reliable marker of cellular senescence (47). Aging MSC lose their fibroblast-like shape and become flatter, longer, presenting a “fried egg” morphology: we reported a significantly increase in size and granularity for oBM-MSC, in line with previously published experiences conducted on replicative senescent MSC (48–50). These changes might be related to alterations in the link between cytoskeleton and cell membrane, due to damages in transmembrane proteins normally assuring this connection (51). Changes in the cytoskeleton composition of MSC have also been correlated with impairment in migration and in the capacity of responding to biological and mechanical signals (29, 52). Several authors postulated about a possible explanation for the increased granularity of senescent BM-MSC correlating it to the increased number of defective cytoplasmic lysosomes containing lipofuscin, organelles, storage particles, and inclusions (53). Regulation of ROS becomes less efficient during aging and an excessive ROS accumulation causes MSC progression to senescence. The induction of this biological process leads to detrimental adaptive responses such as mitochondrial malfunction, autophagy suppression, telomere attrition and protein degradation, contributing to additional ROS production by inducing a positive feedback loop which finally results in a further worsening of cellular senescence (54, 55). Loss of replicative capacity is one of the most important mechanisms related to senescence and it is associated to the overexpression of cell cycle regulators as response to the accumulation of DNA damages (56). Two main pathways for cell cycle regulation involve *p16*/phosphorylated retinoblastoma (*pRb*) and *p53*/*p21* signaling: in our analyses we found an overexpression of *p16* and *p21*, while for *p53* there was not a significantly different expression (57). A similar result was reported by Shibata and colleagues, which found that *p16* and not *p53* was crucial for the induction of cellular senescence, was associated to increased β-Gal expression and that its methylation could cause MSC immortalization (58).

Aging of MSC can cause cell cycle dysregulation and the higher number of cells in S phase has been correlated with upregulation of *p21* and cyclin E21 (59).

Gene expression study revealed profound differences in the two groups of MSC for a series of genes involved in the modulation of inflammatory response. These differences were detected only after stimulating MSC with a combination of cytokines (IL-1β, IFN-α and -γ, TNF-α), which underlines the importance of the surrounding environment on MSC activity (60). We observed increased expression in oBM-MSC for GAL-1, a lectin which has been associated to nuclear factor kappaB (NF-kB) pathway activation and cancer progression, IL-6, a strong activator of acute phase response, IL-8, a chemokine with a specific affinity for neutrophils, TGF-β, a potent inducer of chemotaxis which is able to enhance T-cells activity and TSG-6, a marker of many autoimmune and chronic inflammatory disorders (61–66). Taken together, this data point out how oBM-MSC present profound transcriptome changes compatible with the SASP, showing a paradoxical activity which contributes to inflammation instead of reducing it.

To investigate the effect of senescence on the immunomodulating properties of MSC we focused on macrophages polarization. Macrophages are key elements for the activity of innate immune response and can be found in several states of activation, mostly present in a dynamic and fluid variety of phenotypes, which have been resumed for convenience in the pro-inflammatory M1 and the anti-inflammatory M2 conditions (67). Macrophages can switch from one state to another, especially according to the characteristics of surrounding environment, in terms of cytokines concentration, presence of hypoxia, tissue injury, or organ infection. Each status is characterized by specific markers: M1 markers reflects the pro-inflammatory activity exerted by macrophages while M2 markers amplify signals promoting tissue repair, angiogenesis and cell proliferation (68). In an *in vitro* study on mice-derived MSC, macrophages co-cultured with young MSC expressed M2 markers Arg1 and IL-10, whereas the cells with aged MSC increased M1 related TNF-α. On the other hand, aged MSC increased the migratory ability of macrophages which is a typical property of classically activated M1 macrophages (69).

In our experiments, we focused on MSC effect on M1 macrophages polarization, because of a more important reliability of M1 markers compared to M2, reflecting the heterogeneity of M2 population (M2a, M2b, M2c, M2d) (70). We firstly screened THP-1 derived M1 macrophages for a panel of cytokines to identify significant differences in mRNA expression. Subsequently we conducted experiments focusing on three of these markers (CCL2, TNF-α and IL-6), comparing mRNA expression and protein concentration before and after co-culture with oBM-MSC and yBM-MSC in Transwell systems, to avoid direct cell-to-cell contact and explore MSC paracrine activity. For CCL2 and TNF-α, we reported significantly reduced levels after co-culture with MSC: however, we found that this reduction was significantly inferior after co-culture with oBM-MSC, if compared to yBM-MSC. CCL2 is a chemokine which can attract a wide variety of immune cells (monocytes, macrophages, B and T cells, NK cells, neutrophils), usually produced after tissue injury/infection to promote the extravasation of effector cells to the selected site (71). Increased CCL2 production has also been described in several pathological conditions, mainly autoimmune disorders such as diabetes, atherosclerosis, rheumatoid arthritis, multiple sclerosis and in a wide variety of solid cancers (72). The role of MSC-secreted CCL2 in regenerative models have been studied and it is associated to accelerated angiogenesis and wound healing (73). However, the loss of inhibitory activity on CCL2 production after oBM-MSC and macrophages co-culture that we found, could be a sign of a disrupted regulatory feedback on this axe and could represent one of the mechanisms underlying the “inflammaging” process. TNF-α is considered as one of the main regulators of inflammatory response, determining the activation of important signaling pathways such as NF-κB and mitogen-activated protein kinases (MAPKs) (74, 75). It has been described that TNF-α concentration in culture media (CM) stimulates MSC to activate negative feedback on T-cell proliferation and on TNF-α release by macrophages: according to our data, at least this last ability seems to be significantly less efficient in oBM-MSC (76, 77). The role of IL-6 as a pro-inflammatory cytokine involved in the regulation of both innate and acquired immunity is well known and there are also evidences about a positive correlation with aging and degenerative disorders (78, 79). Despite not being able to demonstrate IL-6 reduction after M1 macrophages co-culture with MSC, we found significantly higher values associated to oBM-MSC, confirming that this cytokine must be considered as an expression of SASP. A relationship between IL-6 secretion by MSC and TNF-α exposure has already been described and could partially explain the mechanism underling our observations (80).

In the last part of the study, we explored miRNA expression in the two groups to detect some of the biological effectors behind the observed differences in MSC immunomodulating activity. MiRNA are small noncoding RNAs that regulate several cellular processes through the epigenetic modulation of genes expression (81). We identified particularly two miRNA, *miR-21* and *miR-193b-3p* that were expressed at higher levels in yBM-MSC. There are evidences pointing out the role of *miR-21* to promote MSC differentiation and tissue repair but also on the enhancement of immunological escape in some neoplastic models (82, 83). *MiR-193b-3p* has been associated to chondrogenesis regulation through histone deacetylase 3 (HDAC3) pathway and to reduced inflammation via NF-kB inhibition in cerebral hemorrhage models (84, 85). Interestingly, HDAC3 has been described as one of the factors regulating the pro-inflammatory secretory activity in macrophages (86). In several pathological model, such as in rheumatoid arthritis, HDAC3 inhibition determined STAT1 inactivation, resulting in reduced levels of some key pro-inflammatory cytokines such as IL-6, IL-8 and CCL-2 (87). We postulate that the increased levels of *miR-193b-3p* found in yBM-MSC could represent one of the factors promoting the switch to the anti-inflammatory M2 condition through epigenetic regulation, which is reflected by the important decrease in the expression of CCL2 and TNF-α seen after co-culture.

In conclusion, our results show that human oBM-MSC present a specific senescent phenotype, different gene and miRNA expression profiles reflecting the acquired SASP and an impaired capacity to reverse macrophages M1 polarization status. All these observations support our hypothesis that senescent human marrow MSC could play a key role in the pathogenesis of “inflammaging” associated with aging, impaired innate immunity and increased risk for chronic diseases.

## Data availability statement

The raw data supporting the conclusions of this article will be made available by the authors, without undue reservation.

## Ethics statement

The studies involving human participants were reviewed and approved by the ethic committee of the CHU Saint-Pierre. Written informed consent to participate in this study was provided by the participants’ or participants legal guardian/next of kin.

## Author contributions

FM: Conceptualization, Data curation, Formal Analysis, Funding acquisition, Investigation, Methodology, Software, Validation, Visualization, Writing – original draft, Writing – review & editing. FC: Data curation, Formal Analysis, Investigation, Software, Writing – review & editing. BS: Investigation, Methodology, Supervision, Writing – review & editing. ND: Investigation, Writing – review & editing. AR: Investigation, Writing – review & editing. NM: Writing – review & editing. DB: Conceptualization, Writing – review & editing. LL: Conceptualization, Data curation, Formal Analysis, Investigation, Methodology, Software, Supervision, Visualization, Writing – original draft, Writing – review & editing.
